# The acceptability of intermittent preventive treatment of malaria in infants (IPTi) delivered through the expanded programme of immunization in southern Tanzania

**DOI:** 10.1186/1475-2875-7-213

**Published:** 2008-10-21

**Authors:** Robert Pool, Adiel Mushi, Joanna Armstrong Schellenberg, Mwifadhi Mrisho, Pedro Alonso, Catherine Montgomery, Marcel Tanner, Hassan Mshinda, David Schellenberg

**Affiliations:** 1 London School of Hygiene and Tropical Medicine, London, UK; 2 Barcelona Centre for International Health Research (CRESIB), Barcelona, Spain; 3 Ifakara Health Research and Development Centre, Ifakara, Tanzania; 4 Swiss Tropical Institute, Basle, Switzerland; 5 National Institute for Medical Research, Amani Centre, Muheza, Tanzania

## Abstract

**Background:**

Intermittent preventive treatment of malaria in infants (IPTi) reduces the incidence of clinical malaria. However, before making decisions about implementation, it is essential to ensure that IPTi is acceptable, that it does not adversely affect attitudes to immunization or existing health seeking behaviour. This paper reports on the reception of IPTi during the first implementation study of IPTi in southern Tanzania.

**Methods:**

Data were collected through in-depth interviews, focus group discussions and participant observation carried out by a central team of social scientists and a network of key informants/interviewers who resided permanently in the study sites.

**Results:**

IPTi was generally acceptable. This was related to routinization of immunization and resonance with traditional practices. Promoting "health" was considered more important than preventing specific diseases. Many women thought that immunization was obligatory and that health staff might be unwilling to assist in the future if they were non-adherent. Weighing and socialising were important reasons for clinic attendance. Non-adherence was due largely to practical, social and structural factors, many of which could be overcome. Reasons for non-adherence were sometimes interlinked. Health staff and "road to child health" cards were the main source of information on the intervention, rather than the specially designed posters. Women did not generally discuss child health matters outside the clinic, and information about the intervention percolated slowly through the community. Although there were some rumours about sulphadoxine pyrimethamine (SP), it was generally acceptable as a drug for IPTi, although mothers did not like the way tablets were administered. There is no evidence that IPTi had a negative effect on attitudes or adherence to the expanded programme on immunisation (EPI) or treatment seeking or existing malaria prevention.

**Conclusion:**

In order to improve adherence to both EPI and IPTi local priorities should be taken into account. For example, local women are often more interested in weighing than in immunization, and they view vaccination and IPTi as vaguely "healthy" rather preventing specific diseases. There should be more emphasis on these factors and more critical consideration by policy makers of how much local knowledge and understanding is minimally necessary in order to make interventions successful.

## Background

Various studies in Africa have shown that intermittent preventive treatment of malaria in infants (IPTi) given at the time of routine vaccinations in the first year of life reduces the incidence of clinical malaria by between 20% and 59% [1-6].

In 2003, when most of these studies were in their early stages, southern Tanzania was in the unique position of having reassuring information on the safety and efficacy of IPTi in the area [1]. As such, it was in a position to address the issues surrounding the development and implementation of IPTi as part of a district-based strategy to control malaria. IPTi was part of the strategic plan of the National Malaria Control Programme which had expressed great interest in obtaining effectiveness information on this promising new component of a malaria control strategy. A five-year programme was established to develop, implement and evaluate a strategy for the delivery of IPTi to five rural districts (approximately 800,000 people). Comparison of process and outcome indicators in areas with and without IPTi provided an opportunity to consolidate its safety profile and to evaluate its impact on (i) the rate of development of antimalarial drug resistance, (ii) perceptions of and compliance with the expanded programme on immunisation (EPI) and (iii) infant health and survival patterns. The effectiveness evaluation has also collected costing data, to produce realistic estimates of cost effectiveness, and included social science studies investigating the acceptability of IPTi (the latter being the focus of this paper). The information gained from this project was intended to shape policy and prepare the way for going to scale with IPTi at the national level. Furthermore, by complementing the efficacy studies conducted within the IPTi Consortium's portfolio of research, this project would serve to guide malaria control programs in other endemic countries.

Before making policy decisions to deliver additional interventions, such as IPTi alongside routine vaccinations, it is essential to ensure that the new intervention does not adversely affect attitudes to, and uptake of, standard EPI interventions. Conversely, it is necessary to know whether the addition of a new intervention to the existing EPI scheme enhances or compromises the perceived value of attending. Furthermore, it is conceivable that IPTi delivered in the context of the EPI may affect existing health- and treatment-seeking behaviour for malaria, as it might be misinterpreted as providing long-term protection similar to that of vaccines. As a consequence, seeking adequate treatment could be delayed. Conversely, increased awareness of malaria as a result of IPTi might enhance the use of preventive and curative measures. It is therefore important to understand people's perceptions of IPTi, and its influence on health behaviour. An additional consideration for the acceptability of IPTi relates to its acceptance and understanding amongst health care providers charged with its delivery, as difficulties at this level are likely to have very major effects on the community effectiveness of the intervention. These issues have been explored and assessed through a longitudinal qualitative study that was part of a five-year project entitled "Community Effectiveness of Intermittent Preventive Treatment delivered through the Expanded Programme of Immunization for Malaria and Anaemia Control in Tanzanian Infants".

Preparation for implementation of the IPTi intervention involved raising awareness through a training programme for frontline health workers, a brand name for IPTi (*mkinge*, which means "protect him/her" in Swahili), adaptations to the "road to child health" (RCH) cards used at health facilities, and specially designed posters to promote the intervention. These posters were developed in a participatory fashion. First a rapid qualitative study investigated potential key messages, images and brand names. Following this, draft posters were designed and pre-tested in similar socio-cultural settings to understand the cultural appropriateness of the content. Posters were then revised until they were deemed acceptable by both project staff and local informants. (This process is described in detail elsewhere [7]). This paper reports on the reception of IPTi and on various contextual factors that influenced this reception.

## Setting and methods

This acceptability study was carried out from February 2005 to April 2007 in Lindi Rural and Tandahimba districts in southern Tanzania. These districts are sub-divided into administrative areas called divisions, with three to 10 divisions in each district and a total of 24 divisions covered by the intervention study. Parts of Tandahimba are on the Makonde Plateau, up to 900 m above sea level. Lindi Rural has hilly areas as well as low-lying plains. There are two main rainy seasons, November to December and February to May, but rain is not uncommon in any month. Malaria is endemic and transmission occurs all year round. The study area has a wide mix of ethnic groups, including the Makonde, Mwera, Yao and many others. Although most people speak the language of their own ethnic group, Swahili is the *lingua franca*. The most common occupations are subsistence farming, fishing and small scale trading. Cashew nuts, sesame and groundnuts are the major cash crops. The main food crops are cassava, maize, sorghum and rice. Most people live in mud-walled and thatched-roof houses; a few houses have corrugated iron roofs. Common water supplies are hand-dug wells that rely on seasonal rain, communal boreholes, natural springs and river water. Most rural roads are unpaved: some are not passable during rainy seasons while others are too steep for vehicles to pass. The public health system comprises a network of dispensaries, health centres and hospitals offering a varying quality of care. Nursing staff are generally responsible for preventive services such as antenatal care, and visits for weighing and vaccination [8].

The study sites were purposively selected to represent areas with specific characteristics, such as proximity to a boundary between IPTi and control divisions, remoteness, and proximity to a main road or the border with Mozambique. Communities living in proximity to boundaries with either intervention or control divisions were also selected to monitor the spread of information beyond the implementation areas.

Data were collected through in-depth interviews, focus group discussions (FGDs) and participant observation carried out by a central team of two trained interviewers and a social scientist that regularly visited and spent time in all the research sites and, following a model that was developed in another study in Uganda [9], data were also collected through a network of eight resident interviewers who lived permanently in the study sites (one interviewer each for the eight different types of site described above – more than eight would have stretched the logistic and management capacity of the central social science team and resulted in more but thinner data). The latter were recruited from among the pregnant women and mothers or caretakers of infants based in the project implementation (six) and control divisions (two) through a process of local advertisements and selection interviews. Job descriptions and person specs were circulated in the study communities and those interested applied in writing (the project needed literate women). Women were short listed and later interviewed by a panel consisting of researchers, village leaders, respected elders and representatives of parents with young children. The resident interviewers were similar with regard to social characteristics: they were all aged between 20 and 40, six were married, one had incomplete secondary school education while the others all had completed primary school. They were trained to carry out systematic observations of behaviours relating to infant health and to make notes of these observations, to informally interview people in their community, and to keep written records of these. Following the example of other projects in the area using community based assistants, they were paid approximately US$25 per month. They were visited, debriefed and interviewed quarterly by members of the central social science team. In addition they mobilized other members of the community for focus group discussions and in-depth interviews that were carried out by the central team. This was greatly facilitated by the fact that they were well integrated in their communities and that community leaders and members were supportive of their role in the project. The use of local resident interviewers enabled detailed, long-term participant observation of health behaviour and the spread of information about the intervention. This enabled the collection of detailed first-hand data on practical situations that would otherwise have been difficult to access.

In total, eight rounds of visits to the resident interviewers were made and a total of 70 debriefing interviews with them recorded. An additional 96 in-depth interviews and 44 FGDs were conducted with mothers of young infants, health workers and other members of the community. These interviews and the FGDs were flexible and topics changed as the study progressed, enabling relevant themes and topics that emerged to be followed up. However, some questions were repeated in order to collect information on change in attitudes and practices. In addition to the above techniques, one of the team (AM) carried out an ethnographic study, involving participant observation in health centres and communities, in two of the study areas between October 2006 and July 2007. Some observational data from this study are also used in this paper.

All interviews and FGDs were recorded digitally and transcribed and these transcriptions, together with the observational notes were coded and analysed using NVivo (software for qualitative data analysis). A grounded theory approach was used to inform coding and subsequent analysis, with later interviews and observations building on pertinent areas of inquiry revealed in earlier interviews and field notes. Grounded theory is a systematic research methodology that enables the generation of theory from qualitative textual data. The hallmarks of grounded theory are theoretical sampling, constant comparison, and theoretical saturation. Analytic memos were written for the duration of data collection and analysis, and this guided initial interpretation. Interviews were coded systematically using inductively generated codes to identify important themes, with coded sections compared within and across cases to generate higher order generalizations [10-12]. Analysis of the coded data in the Nvivo project (i.e. the data coded for the themes and topics relevant to the project) did not reveal any substantial differences between data from the different sources, methods or sites relating to the topics discussed in this paper. While many hours' worth of interview text was analysed, short quotations have been selected for this paper to illustrate the main findings.

### Ethics clearance and confidentiality

The main IPTi project of which this acceptability study was a part was conducted within the framework of the IPTi Consortium  and received ethical approval from the local and national institutional review boards of Ifakara Health Research and Development Centre, Tanzania, the National Tanzania Medical Research Co-coordinating Committee, the Tanzania Commission for Science and Technology, the London School of Hygiene and Tropical Medicine and the Swiss Tropical Institute. Verbal informed consent was sought from all participants and recorded at the time of interviews or focus group discussions.

All digital recordings and transcripts were stored on secure computers to which only project staff had access. Recordings were deleted from recorders once they had been uploaded. All participants were identified through identification numbers.

## Results

### Traditional preventive practices for infants

Traditional preventive practices for infants are common in this area, as in many other parts of Africa. Illnesses that are perceived as being prevented through traditional treatment include convulsions (*likonde*). Treatments vary from herbal decoctions and rubbing herbal powders into incisions on the head to amulets made up of herbs and scraps of paper containing writings from the Koran. Much of the treatment is in the form of preventive amulets, for example to protect the child against witchcraft or evil spirits, or from the weakness that results from being touched by the father if he has been unfaithful (*kumtimbangila mwana).*

### Reasons for EPI clinic attendance

EPI clinic attendance was relatively routinized and women tended to have vague notions about vaccination being beneficial. They often said that it was "good" for the child. When they were pushed to be more specific, they often said they attended the clinic in order to "know about the child's development". Sometimes women did go to the clinic for a particular reason, such as weighing, health education, or getting "injections and drops". A few women were able to name specific vaccinations, but most simply distinguished between "injections" and "drops", or between "injections in the arm" and "injections in the thigh". The purpose of these injections and drops was described in very general terms as "protection" (*kinga*).

Some mothers said they felt obliged to attend the EPI clinic because they feared that if they were perceived as non-adherent, staff might be unwilling to help them when they came for illness or delivery. They claimed that staff would know that they had missed EPI clinic visits from the "road to child health" (RCH) card and get angry.

*If a child is sick you will not be helped at the hospital if you haven't taken him for vaccination. This is not good because it will take too much of your time when accessing services*. (Mother)

Underlying this was a more general notion, also reported in relation to EPI clinic attendance in Mozambique [13], that adherence was some sort of civil obligation.

Another significant reason for attending clinic was social: it was somewhere that women could go to get away from the tedium of farm work and domestic chores and meet, chat and socialize with peers. It was a rare opportunity to dress up and "go out".

*[Some women] enjoy going to clinic because they get an opportunity to interact with other women and relieve themselves from farming activities. Others say that it is the day for appearing smart [well dressed]*. (Resident interviewer)

The main reasons for missing the EPI clinic were practical: heavy rain, farm work, distance, illness, (reported) absence of the doctor, or information about the clinic being closed or not having the necessary vaccines. The perceived side effects of vaccination, in particular of the injections (swelling, fever, abscesses) were commonly mentioned as a negative aspect of clinic attendance, and this was also an important reason for non-adherence.

Opinions about the health staff varied widely. Some reports were positive: health workers were friendly, helpful and informative; others (the majority of evaluative comments) were negative: health workers were rude, badly behaved and uninformative. Negative reports included a doctor who was often drunk, a nurse who refused to help in emergencies, and a clinical officer who refused to attend to women in labour and who sold clinic medicines in the village. Complaints about long waiting times, overcrowded waiting rooms, and shortages of drugs were also common. Respondents' reports were often mixed, with positive aspects or experiences offsetting more negative ones. For example, in the discussion of the case of the clinical officer above, focus group participants also said that they were satisfied with the information about IPTi that they were given at his clinic.

Respondents recognized that there were sometimes good reasons for the poor service. The most commonly mentioned factors were staff shortages, lack of drugs, and lack of refrigeration complicating the logistics of vaccination. Some women also admitted that mothers sometimes did not listen to the nurses and recognized that this was perhaps a legitimate reason for the latter becoming irritated and angry.

The reasons for individual non-adherence to immunization were sometimes complex. For example, a woman might miss a clinic visit for entirely legitimate reasons and then fear to go back for subsequent visits because she is afraid that the nurse will be angry with her. Sometimes structural factors outside the control of individual mothers or clinic staff were involved. For example a woman who does not attend the EPI clinic because she has heard that there are no vaccines. Enquiries at the clinic reveal that the lack of vaccines is due to there being no refrigeration, which is a result of the electricity company cutting off the supply to the clinic because of late payment of bills.

### Community knowledge and awareness of IPTi

During the first six months of implementation, respondents hardly mentioned IPTi (*mkinge*) when asked about services provided to children at the EPI clinic. Both direct and indirect data collected in subsequent interviews and FGDs showed that awareness gradually increased as implementation expanded, until most respondents in the six implementing divisions either mentioned *mkinge *spontaneously or at least said they had heard about it when prompted. However, even when implementation was well underway there were still some who claimed that they had not heard of *mkinge *at all, or who had heard about it but did not know what it was for. Even relatively late in the intervention some respondents were still reporting that *mkinge *was given to children to "cool down" the fever caused by vaccination. Some participants claimed that they had not heard of *mkinge *even though their clinic cards showed that their children had already received it. Awareness that *mkinge *specifically prevented malaria increased, although by the end of the study many respondents still thought that it "protected" against ill health generally rather than malaria specifically.

It is clear from the interview, FGD and ethnographic data that neither vaccination nor *mkinge *were generally discussed outside formal health information sessions in the clinic. In fact, it seems that as long as women do not perceive problems they do not generally discuss health issues relating to their children at all. As one resident interviewer put it, referring to mothers generally:

What they care about is whether their child is eating well and playing [i.e. behaving normally, healthily]. If the child isn't sick then the mother doesn't talk of hospital issues.

The fact that information about *mkinge *did not feature in the discussions with resident interviewers and other mothers of young children in non-implementing areas a year after the start of implementation illustrates how slowly this information percolated through the community. Respondents in these areas did start to mention *mkinge *towards the end of the implementation, but only when probed.

### Sources of information about IPTi

Clinic staff and RCH cards were a much more important source of information about IPTi than the specifically designed posters that were displayed at health facilities. In fact, many people who were asked said they had not noticed the posters at all.

There were two very different responses to questions about the role of health care staff in the dissemination of information about IPTi. Some respondents were positive about information on IPTi received from health staff during clinic visits; others complained about receiving insufficient information.

*My child received *mkinge, *but I don't know what it cures or means. I only remember that they gave him that tablet and we left without being told anything*. (Mother)

Common complaints included insufficient explanation of possible side effects of *mkinge*, the age at which children were eligible to receive *mkinge*, how long a child would be protected after receiving *mkinge*, and how a child should be managed in case of malaria/fevers after receiving *mkinge*. This perceived information gap did not only apply to *mkinge *but also EPI and antenatal services more generally. Discussions with all resident interviewers and FGD participants in five out of eight divisions included complaints about unfriendly health workers and insufficient information provided to mothers at clinic. Perceived unfriendliness of staff and lack of information were related in that fear of staff reactions often prevented mothers from asking for information.

Respondent: I asked other women who had been given earlier.

Interviewer: Why didn't you ask the nurse?

Respondent: We are afraid of her; we can't ask; we just do whatever she instructs us to do.

Women often said that they were afraid to ask questions in the clinic because other mothers might laugh at their ignorance.

### Attitudes to IPTi with sulphadoxine pyrimethamine (SP)

It is not easy to separate attitudes to IPTi, IPTp (Intermittent Preventive Treatment in Pregnancy), EPI and SP in the data. Immunization delivered through EPI was generally acceptable to mothers of young children, health workers and the wider community, and taking infants for vaccination had become routine behaviour for most mothers in the study communities. Respondents were already generally familiar with SP, first as a widely available anti-malarial and then more recently as the drug used for IPTp. IPTp was generally regarded positively and, in addition to preventing illness in general and malaria in particular, it was thought to "increase blood" (probably referring to the effect on anaemia) and to keep the baby in the womb healthy. IPTp and IPTi were often seen as related, with the former protecting the baby in the womb and the latter continuing that protection after birth. Most women said that they had not experienced any side effects from IPTp.

Respondents who had heard about IPTi were generally positive in their response. Perception of the benefits ranged from promoting general well being and preventing undefined illness to specifically preventing malaria. When questioned explicitly, respondents generally said that they did not think that IPTi completely prevented malaria, but that it only reduced the frequency or severity of the illness.

It [IPTi] doesn't mean that the child won't get sick at all; it just makes it less severe. (Resident interviewer)

This fits very well with both their perception of the partial efficacy of both vaccination and traditional preventive treatment for infants as well as with a slightly fatalistic view of illness causation.

*If god decides a child will fall sick with malaria, even if he has taken *mkinge *[then he will]. But protection is very important, and if you are lucky enough, after taking *mkinge *you may not fall sick*. (Resident interviewer)

Some women reported that their child had not had fever/malaria or any serious side effects since receiving IPTi.

*Once a child uses that *mkinge, *the body temperature doesn't rise. My child plays well after getting that mkinge*. (Mother)

There were relatively few complaints about IPTi per se, and most of these related to mild perceived side effects. Rashes and other skin lesions were the most commonly mentioned negative side effects, but fever and swelling were also mentioned. These negative comments were usually in the form of second-hand descriptions, with relatively few first-hand accounts:

*I took my child for vaccinations at the dispensary, and he was given a tablet on a spoon. The nurse said: this is *mkinge *and don't give your child another drug within 2 weeks. Within two weeks of using it the child developed skin rashes over the whole body*. (Mother)

Rumours about side effects of SP were mentioned in some focus groups. These rumours were usually about people's skin "falling off" after taking SP and were derived from media reports of Stevens-Johnson syndrome. There was, however, the odd more specific report:

*A school child in class 5 died recently at the hospital. They had given her those Fansidar tablets. I don't know if they gave her three tablets instead of the two that she should have had. So her skin peeled off and she died*. (Mother)

Rumours relating to SP were sometimes linked to rumours about mass drug administration campaigns for filariasis and trachoma treatment/prevention in the study areas, which some respondents claimed had been initiated by foreigners in order to depopulate the country. During the ethnographic fieldwork, rumours spread that foreign scientists had invented a mosquito that was going to spread malaria at an alarming rate, and some villagers suspected that combination therapy for malaria had been introduced from outside for experimental reasons. However, most respondents discarded such rumours, claiming to trust the government.

### The administration of IPTi

There were also various complaints about IPTi that did not relate to side effects. The most important of these related to the way that IPTi was administered in the clinic, using shared spoons and cups, which many women thought was unhygienic.

Although a household survey carried out as part of the main intervention study in 2006 (unpublished data) found that mothers reported giving only 6% of the IPTi doses at home, there is evidence from the in-depth interviews, but especially from the observational data, that it was more common for clinic staff to give mothers SP tablets to administer the IPTi at home, in spite of clear instructions not to do this. In addition to mothers' concerns about hygiene, some staff saw the delivery of IPTi as an extra burden and found it easier to get the mothers to administer it themselves. There were also structural constraints, such as shortages of water, which impeded clinic administration, and staff felt that home administration was better than missing treatment. To some extent home administration was the result of collusion between health staff and mothers. The ethnographic data show that some mothers administered the IPTi correctly at home. However, others did not: some forgot, some thought that the IPTi was meant to "cool" the fever caused by vaccination and only gave it if the child had a fever, and others administered it according to their own idiosyncratic scheme. However, mothers were generally in favour of their child being given IPTi in the clinic.

### Interaction between IPTi and EPI

There is no evidence in the data from this study that IPTi had a negative effect on attitudes to EPI or that it had any negative impact on EPI adherence. Nowhere in the recorded interviews or focus group discussions did anyone admit that they had made less use of EPI or had considered making less use of EPI due to their child having received IPTi. Nor were there any indirect references that could be interpreted as referring to a negative impact on EPI attendance or attitudes to EPI. Moreover, none of the observational data, either from the local interviewers or the participant observation, suggested reduced EPI attendance as a result of IPTi.

IPTi was not generally perceived as a form of immunization against malaria (i.e. when explicitly asked, none of the respondents said that they thought that a child who had received IPTi would no longer be susceptible to malaria) and it did not appear to have any serious negative impact on self-reported treatment seeking for febrile illness or on attitudes to or use of other forms of malaria prevention (which in the study setting were mainly bed nets).

When asked explicitly, women generally insisted that their treatment seeking behaviour would be the same if their child had fever, whether the child had received *mkinge *or not. In both focus groups and individual interviews mothers were presented with hypothetical scenarios of two identical children with identical febrile symptoms, but only one child had received IPTi. They were asked how they would respond to the illness. No one reported different treatment-seeking responses for the child who had received IPTi.

However, an examination of the more indirect references to efficacy reveals that some mothers did assume that their children would no longer get malaria/fever after they had received *mkinge*. For example, one resident interviewer reported:

*Some mothers expect *mkinge *to prevent malaria in their children, so they don't understand why their children suffer malaria when they are protected*.

While not very common, complaints by mothers about their children contracting malaria after they have received *mkinge *are scattered throughout the data. Even one of the resident interviewers said that her child got malaria within a month of the first dose of IPTi. She said she would not let her child have IPTi again if she was given a choice. Another resident interviewer said:

*I don't see the point (of *mkinge*) because my child got malaria two weeks after receiving it. I was not given adequate information about whether or not he would still get malaria.*

### Influence of men on decision-making

Although fathers were formally the final decision makers relating to health seeking behaviour, respondents agreed that men generally do not intervene in decisions about infant and child health. In practice it is the mother's responsibility for taking the child to the clinic and mothers have relative freedom in deciding on this without having to always consult husbands or other relatives such as mothers-in-law.

*It is normal to take a child to clinic without notifying him because he knows that we [mothers] are supposed to take a child to clinic each month*. (Mother)

*The husband is involved but the person who looks after the child is the mother. When the child falls sick men leave the entire burden to the woman to take the child to the hospital. Therefore the woman is the one who knows what is going on with the child*. (Resident interviewer)

A few fathers were critical about IPTi. One resident interviewer reported hearing a man being angry with his wife for agreeing to give their child SP when the child was not sick. The man had said:

Mothers just accepted drugs that are meaningless, because we are used to a child being given a drug when he/she is sick and not for prevention. Had my child been given the drugs to use at home, I would have thrown them away.

It is unclear how much influence these men had on actual decision-making, but it is clear that in general men were not a major factor in adherence to either EPI or IPTi.

### Social interaction in the clinic and the wider social context

On a more general level, the reception of IPTi was influenced by local social relationships. The relationship between health workers and mothers is hierarchical, with mothers dependent on health workers for services and generally adopting a passive role when they interact with them in a clinic setting. As a result, even when women do go to the clinic for a specific purpose they often end up not getting what they want. During the ethnographic study, for example, one woman who wanted to know her child's weight was not told after the child had been weighed and, although she really wanted to know the child's weight, didn't dare to ask. Another woman, who went to the clinic expecting vaccination after the weighing, was told by the nurse that it was not time for the child's vaccination, so she went home without enquiring further. Similarly, another mother reported how, but for the intervention of a friend, her infant would not have received the IPTi that she had come to the clinic to obtain:

*My child was about to leave the dispensary without getting *mkinge. *They weighed my baby and gave the card back to me without asking me to wait for *mkinge. *The card was also not retained for vaccination. I would have left without my child getting vaccinations and *mkinge, *had I not met my friend who asked me to join the queue. Thereafter my child was vaccinated before I was given *mkinge *to give my child at home.*

When women did not receive what they wanted or expected in the clinic, they did not ask why. They frequently admitted that they did not understand something that the health worker said, or that they wanted to ask a question, but didn't dare to ask, usually because they thought that the health worker would get angry or ridicule them, or because they thought that the other mothers would laugh at them. This was not merely an unfounded perception, as staff did get angry with mothers and women were laughed at, and women's passive behaviour was exacerbated by the insecurities inherent in the hierarchical nature of the clinic setting. In addition, because patients and health workers came from the same relatively small rural communities, the relationships, and the social tensions inherent in those relationships, spilled over into the clinic setting and influenced the interactions there as well.

Taking the wider context into account also served to explain and contextualize some of the negative comments about health staff. Observing health workers in the community setting outside their official role in the clinic revealed that the work of many clinic staff did not end when the clinic closed. Neighbours, relatives, friends, and community members consulted them at all times of the day or night if there was an en emergency or a need for health care, or if assistance was required for a delivery. Knowledge of specific incidents made it possible to understand why a health worker did not appear to be working as hard as she should, or was found dozing during work: she had been up all night assisting with a delivery.

## Discussion

In this area, immunization was generally acceptable and was routine behaviour for most mothers. IPTi benefited from this situation by fitting into already established patterns of behaviour in an unobtrusive way, and from the resonance with traditional preventive practices. Although most mothers did not have detailed knowledge of immunization, they generally interpreted it as something beneficial. Mothers saw weighing as an important health-care activity in itself, and this appears to have had a positive effect on EPI clinic attendance. Clinic attendance was also a social event that gave women a break from the monotony of domestic chores and farm work and an opportunity to dress up and meet friends. These factors are all similar to the findings of a study in Mozambique [13].

When women defaulted, this was largely a result of practical factors. However, the reasons for non-adherence were sometimes multiple and complex, and also involved structural factors outside the control of individual mothers or clinic staff. This suggests that interventions to improve coverage of and adherence to EPI and IPTi (but also other interventions) should take wider structural impediments into account. While it is not feasible in the short or medium term to address the broad structural constraints described by authors such as Paul Farmer as "structural violence" (poverty, inequality) [14] it is feasible to change the more local structural constraints identified in this study (e.g. water and electricity supply to health centres). There is also scope for emphasising or enhancing women's experience of those aspects of clinic visits that they perceive as important, even though they may be less essential from a public health point of view. For example, more emphasis could be placed on the weighing of children, and this could be better organized, and more could be made of clinic days as social events.

Two of the major concerns relating to the reception of IPTi linked to EPI have been that new additions to EPI might negatively influence people's attitudes to and uptake of immunization, and that people might misunderstand IPTi as immunization against malaria and as a result neglect other preventive measures. Regarding the former, there is no evidence in this setting that IPTi had any negative impact on attitudes to EPI or that it had any effect on EPI adherence. Regarding the latter, people in this study, as in the Mozambique study [13], did not generally think that having received IPTi meant that their child would no longer get malaria. This was not because they were aware of the limited protection offered by IPTi, but rather because they viewed all prevention, including both traditional preventive practices and biomedical immunization, as partial – attenuating rather than completely preventing disease. This also resonated with a fatalistic view of the cause of some illnesses: they "just happen" (these are the illnesses described as "naturalistic" or "caused by god" in traditional African aetiologies) [15]. However, this does not mean that IPTi did not have any negative effect on EPI related attitudes and behaviour, just that there was no clear evidence of this in the qualitative data in this study. Also, even though there was no evidence in the data that IPTi had affected treatment seeking behaviour for febrile illness in infants, this was based on self-reporting and hypothetical case scenarios, and it is possible that some women did respond differently in reality.

There is indirect evidence from the data that some mothers did not expect their children to get malaria after they had received IPTi, and that in some cases this did affect treatment seeking. This is worrying and should be a focus in education campaigns accompanying implementation. In particular, information campaigns could be designed to fit more closely with prevailing and widely held ideas about health prevention. As long as local perceptions of efficacy are not directly harmful to the intervention, it is not necessarily a problem that they are not entirely accurate from a biomedical point of view.

One clear complaint that many mothers had about IPTi related to the way it was administered. It was not so much the crushing of tablets and giving it to the infant on a spoon that troubled them, but what they perceived as the unhygienic sharing of cups and spoons. This serves to emphasize the importance of developing a formulation for IPTi that is more suitable for infants.

Also relating to the administration of the drug, there was evidence that health workers gave the SP to mothers to administer at home, although when asked explicitly they always denied this. It is impossible to say precisely how prevalent this was: much of the evidence for this was derived informally during participant observation, and all those involved had an interest in not revealing this to the researchers. As with other issues discussed here, there were positive and negative aspects to this, and also wider structural factors that need to be taken into account. To some extent home administration was the result of collusion between health workers who wanted to reduce their work burden or make sure that children did not miss a dose due to water shortages in the clinic, and mothers who wanted what they perceived as being best for their child. There is clear evidence of mothers taking the SP home and not using it as recommended, but there is also evidence of mothers implementing the IPTi at home in the proper manner. The fact that clinic staff and mothers colluded in the home administration of SP reminds us firstly that health behaviour in practical situations often deviates from the intentions of health planners and the knowledge that health staff have of what they are supposed to do; and secondly that adherence can sometimes be more adequately achieved by going along with what users do anyway and trying to adapt this practical behaviour in subtle ways, rather than trying to counter it.

IPTi was not widely discussed in the community and information about it percolated through the local communities very slowly, in spite of a well designed and implemented information campaign. However, this should not be taken to mean that information does not travel rapidly and efficiently through such rural communities: there is ample evidence of the efficiency of *radio trottoir *[sidewalk radio], as the informal circulation of information is referred to in francophone Africa [16]. Some kinds of information (for example rumours about blood stealing or about the sterilizing effect of vaccinations) can spread across vast areas in a matter of days [17-19]. The fact that people apparently did not pay much attention to the IPTi posters and did not discuss IPTi outside the clinic, and the fact that information about IPTi spread through the community relatively slowly, can be interpreted in two ways. On the one hand, it could be concluded that the information strategy did not work, and that people were not interested enough in the intervention to discuss it. On the other hand it could be argued that IPTi fitted seamlessly into an already existing and widely accepted intervention and set of underlying assumptions about prevention, and the fact that it wasn't widely discussed simply reflects the success of this integration. If people's perceptions in the intervention areas had been overwhelmingly negative then rumours would probably have spread rapidly via *radio trottoir*.

These aspects of the circulation of health related information through local communities have implications for health education and information provision more generally. For example, studying the way in which information travels through the informal channels of *radio trottoir *and the form and nature of the messages thus transmitted could suggest ways of using similar mechanisms to transmit public health messages. Also related to the provision of information, the fact that so many participants claimed that the child's RCH card was an important source of information suggests the possibility of developing individual sources of information that are more personalized than leaflets that patients can throw away.

Experience with successful interventions in communities that are generally positive though not very knowledgeable about the intervention, and where success is based on routinization and vague notions of health, should lead to discussions about how much and what kind of information is minimally necessary to ensure the success of health interventions. Perhaps the assumption – that the more information that is given the better – is not the right one. It should also lead to a questioning of the assumption that the long-term success and sustainability of interventions such as immunization require active demand rather than just passive acceptance [20,21].

It is clear that the relationship between patients and health workers in these rural communities is ambivalent and that social relationships spill over from the community into the clinic setting where neighbours suddenly have to take on the roles of health provider and patient. A study of the demand for malaria treatment and prevention in Tanzania revealed that mothers often have the necessary knowledge to seek appropriate care for their child, but are effectively disempowered in the clinical encounter by aggressive staff who assume they are ignorant [22]. These relationships and the contexts in which they are embedded should be studied further in order to improve relationships and communication within the clinic.

In the shorter term there are various possibilities. One is to provide staff with better training. This by itself would not be sufficient, though, and would also require supportive supervision and the development of a problem-solving attitude among both health staff and their supervisors. There would probably be more supportive supervision for IPTi staff if it was national policy and fully adopted by the EPI programme. Any intervention aimed at health staff would need to take into account the additional work burden due to health workers feeling morally obliged to provide care outside their formal work environment and working hours. Also, health facility boards could play a role. These are supposed to be a mechanism to improve health care services (involving influential community members who are supposed to have some authority over the health staff), but they are poorly understood and not very effective.

However, in the final instance substantial improvement would require a much broader programme aimed at developing a culture in which health staff respect their clients and make their clients' best interests their primary concern, and in which patients realise that they have rights in this respect and learn to demand the information or the services that they have a right to.

## Conclusion

In this setting, IPTi delivered together with EPI was generally acceptable. Acceptability was related to prior routinization of EPI and resonance with traditional practices. Non-adherence was due largely to practical, social and structural factors, many of which could easily be overcome. These factors include local social relationships and the way in which they are reproduced in health care settings.

Local priorities should be taken more into account, even though they may not be the highest public health priorities, as long as they are not harmful and can facilitate the uptake of interventions (for example, more emphasis on weighing and socialising during clinic visits, and presenting IPTi as "healthy" rather than over-labouring the prevention of malaria). Related to this, there should be more consideration from a practical point of view about the minimum knowledge and understanding required to make interventions successful, rather than simply assuming that more is better.

The longer term successful implementation of IPTi will require either the development of an acceptable formulation for infants (e.g. drops), the development of more acceptable means of administering the tablets, or the acceptance of home administration by involving (and empowering) mothers as active collaborators. This could also be important if a multi-dose regime is used for IPTi with alternatives to SP.

On a more general level, what is needed is the development of a culture of respect between health worker and patient and the empowerment of patients to stand up for their rights.

## Authors' contributions

RP played a major role in the design of the acceptability study, the development of the instruments, the supervision of the data collection, and the interpretation of the data. He also took the lead in writing the paper. AM participated in the study design and the development of the data collection tools, he played a major role in the collection of the data and he helped to interpret the data and write the paper. MM Contributed to data collection and the interpretation of results and commented on the manuscript. CM contributed to the design and management of the Nvivo software project used to manage and analyse the data, to the interpretation of the data and to the writing of the paper. JS and DS provided overall supervision of the research, input on the study design and data collection, and contributed to analysis and writing this manuscript. HM, MT and PA provided technical input to the design of the overall study and commented on the manuscript.
